# New Tricks by an Old Dog

**DOI:** 10.1155/2008/384219

**Published:** 2008-09-18

**Authors:** Thomas Forst, John Wahren

**Affiliations:** ^1^Department of Endocrinology, Johannes Gutenberg University, Parcusstrasse 8, 55116 Mainz, Germany; ^2^Department of Clinical Neuroscience, Karolinska Institute, 171 76 Stockholm, Sweden

It is generally recognized that the connecting peptide (C-peptide) of proinsulin fulfills an important function in the biosynthesis of insulin. It brings together the A- and B-chains such that the initial folding and interchain disulfide bonds can be formed. Evolutionary considerations suggest that a length of approximately 30 residues for the connecting segment, as is the case for human C-peptide, is optimal for the efficient further processing of the molecule (i.e., its cleavage into insulin and C-peptide). Following this, the two are stored in secretory granules and eventually coreleased into the circulation. Because of its intimate connection to the insulin biosynthesis, C-peptide has been used as a marker of insulin secretion. As such, it has contributed importantly to our understanding of the pathophysiology of several metabolic disorders, notably type 1 and type 2 diabetes.

The possibility that C-peptide may possess biological effects of its own was considered but received relatively little attention at the time of its discovery in 1968. No detectable influence on glucose metabolism or on lipolysis of isolated fat cells could be observed. In the absence of any insulin-like effect by C-peptide in isolated cell systems or when administered to healthy individuals, it was concluded that C-peptide was without biological effect other than its role in the biosynthesis of insulin; for a review see [1]. Consequently, C-peptide as a bioactive peptide left the scientific limelight and the interest was focused instead on its usefulness as a marker of insulin secretion.

It was not until the early 1990s that direct C-peptide effects were re-evaluated. A series of studies was undertaken involving administration of the peptide in type 1 diabetes patients, who lack C-peptide [2]. This proved a useful approach and it became apparent that replacement of physiological concentrations of C-peptide in this patient group results in significant amelioration of diabetes-induced abnormalities of regional blood flow as well as improvements in peripheral nerve and kidney function. These surprising findings, subsequently confirmed and extended by several laboratories, prompted a renewed interest in C-peptide as a bioactive peptide in its own right. Since then, a steadily increasing number of reports on new aspects of C-peptide physiology have been presented. Today, a vast body of scientific evidence is available comprising in vitro studies of the peptide's membrane interaction and cellular effects, in vivo studies in animal models of type 1 diabetes defining C-peptide's influence on functional and structural abnormalities of the kidneys and the peripheral nerves as well as clinical trials on nerve and kidney function in patients with type 1 diabetes, all of which attest to a wide spectrum of physiological effects being mediated by C-peptide. In addition, the findings provide a basis for the notion that C-peptide administration, in combination with regular insulin therapy, may be beneficial in the prevention and treatment of microvascular complications of type 1 diabetes.

In the present, special issue of *Experimental Diabetes Research*, most of the recent developments in C-peptide research are being reviewed including an authoritative review of the history and diagnostic aspects of C-peptide. A highly qualified attempt is made to sort out the multitude of intracellular effects of C-peptide, seemingly contradictory when studied in different cell systems and under varying experimental conditions. Perhaps the most compelling end effect of C-peptide is its stimulatory influence on the microcirculation in a number of tissues, achieved via both activation and induction of endothelial nitric oxide synthase. These events are reviewed as are the beneficial effects of C-peptide and its C-terminal hexa- and pentapeptide segments on the diabetes-induced reduction of red blood cell deformability. A possible stimulatory effect by C-peptide on glucose uptake is discussed on the basis of both in vitro experiments and findings in type 1 diabetes patients. It is, however, noted that interpretation of such results is confounded by the recent observation that C-peptide may elicit disaggregation of insulin hexamers, thereby augmenting the availability of bioactive insulin monomers [3].

C-peptide and its influence on renal physiology, particularly tubular function, are discussed. Likewise, C-peptide effects on the peripheral and central nervous system are reviewed. Much new and valuable information in this central area of C-peptide research has been presented from Anders Sima's laboratory. The comprehensive findings now point towards a need for clinical trials and the current situation regarding clinical studies in patients with diabetic neuropathy is described. Finally, the possibility that C-peptide may serve as a mediator in the development of atherosclerotic lesions is discussed. Is the peptide guilty as charged or wrongly accused? Only future studies can tell but, attesting to the rapid developments in the field of C-peptide physiology, a study just published reports that physiological as opposed to elevated concentrations of C-peptide serve to diminish hyperglycemia-induced vascular smooth muscle proliferation [4].

The purpose of this issue is to provide an update of our understanding of C-peptide physiology and the role of C-peptide deficiency in the development of microvascular complications of type 1 diabetes. Clearly, there is much more to be learned about C-peptide. Identification of a receptor or the mechanism whereby C-peptide interacts with the cell membrane has a high priority. On the clinical side, further trials of long duration are needed to define the possible role for C-peptide, together with insulin, in the treatment of type 1 diabetes. A major obstacle for extended clinical trials has been the lack of GMP-produced C-peptide suitable for human use. It is hoped that the evidence summarized in this issue will convey the urgency with which clinical studies are needed and stimulate the interest of funding organizations and the pharmaceutical industry to become involved in this rapidly developing field.

*Thomas Forst* *Thomas Forst*

*John Wahren* *John Wahren*
